# Reproducibility and Consistency of *In Vitro* Nucleosome Reconstitutions Demonstrated by Invitrosome Isolation and Sequencing

**DOI:** 10.1371/journal.pone.0103752

**Published:** 2014-08-05

**Authors:** Colton E. Kempton, Justin R. Heninger, Steven M. Johnson

**Affiliations:** Department of Microbiology and Molecular Biology, Brigham Young University, Provo, Utah, United States of America; University of Regensburg, Germany

## Abstract

Nucleosomes and their positions in the eukaryotic genome play an important role in regulating gene expression by influencing accessibility to DNA. Many factors influence a nucleosome's final position in the chromatin landscape including the underlying genomic sequence. One of the primary reasons for performing *in vitro* nucleosome reconstitution experiments is to identify how the underlying DNA sequence will influence a nucleosome's position in the absence of other compounding cellular factors. However, concerns have been raised about the reproducibility of data generated from these kinds of experiments. Here we present data for *in vitro* nucleosome reconstitution experiments performed on linear plasmid DNA that demonstrate that, when coverage is deep enough, these reconstitution experiments are exquisitely reproducible and highly consistent. Our data also suggests that a coverage depth of 35X be maintained for maximal confidence when assaying nucleosome positions, but lower coverage levels may be generally sufficient. These coverage depth recommendations are sufficient in the experimental system and conditions used in this study, but may vary depending on the exact parameters used in other systems.

## Introduction

Nucleosomes play an important role in gene regulation. Eukaryotic genomes are highly compacted and nucleosomes are the most basic of the many levels of compaction. Nucleosomes are formed when 147 base pairs (bp) of DNA wrap about 1.6 times around a histone octamer [1]. Gene regulation in eukaryotes frequently begins at the transcriptional level with trans-acting factors binding the DNA. A nucleosome's genomic position and which DNA bases are facing towards or away from the nucleosome, often described as translational and rotational setting respectively, can affect many important processes by influencing the availability and function of binding sites encoded in the DNA [2]. Therefore, nucleosomes and their positions on the DNA are the first level of eukaryotic gene regulation.

What influences and ultimately determines a nucleosome's position within the genome is complex with many groups actively researching this question. Some of the factors that influence a nucleosome's position include the underlying DNA sequence, chromatin remodeling factors, DNA binding proteins, transcription factors, and even neighboring nucleosomes [3]. Many experiments have been done *in vitro* and *in vivo* to examine how these factors affect positions of nucleosomes and gene regulation *e.g.* [4], [5], [6], [7], [8], [9].

*In vitro* nucleosome reconstitution is done by mixing naked DNA fragments and isolated or recombinant histone octamers together in a high-salt environment. While the salt is slowly dialyzed out of this solution, spontaneous interactions between the DNA and histone octamers result in the formation of nucleosomes on DNA sequences that are most thermodynamically favorable [10]. One purpose of these experiments is to observe and define the influence that underlying DNA sequence has on nucleosome formation. This is done by observing the positioning and occupancy of reconstituted nucleosomes on defined DNA sequences or even whole genomes. Several groups, including ours, have used this method to demonstrate that nucleosome occupancy and positioning is highly dependent, at least *in vitro*, on the nature of the underlying DNA sequence [6], . The proclivity of a nucleosome to form and the precise positioning of a nucleosome on a DNA sequence can be two separate, yet often conflated functions of the underlying DNA sequence. In reality, these separate functions can be directed by individual elements within the DNA forming the nucleosome core and linker regions [6].

While several sequences have been shown to be highly consistent in their ability to attract and precisely position nucleosomes (e.g. 601, sea urchin 5S, container site)[6], [8], [11], [12], some researchers continue to doubt the reproducibility of *in vitro* nucleosome reconstitution across less well defined sequences and even the reproducibility of positioning across experiments. These criticisms stem from the fact that multiple nucleosome positions can be adopted on DNA fragments that are greater than 147 bp or even on a DNA fragment of only 147 bp [10], [13], which is then often interpreted to mean that *in vitro* reconstitution experiments are inconsistent in their outcomes and hence irreproducible and unreliable. Here we present evidence that *in vitro* nucleosome reconstitution experiments on plasmid DNA assayed by micrococcal nuclease (MNase) digestion and high-throughput sequencing (MNase-seq) are both reproducible and highly consistent allowing confident analysis of both nucleosome positioning and occupancy using this technique.

## Results and Discussion

### I*n vitro* nucleosome reconstitutions on linearized plasmid DNA and invitrosome DNA sequencing

In order to address the question of consistent positioning and occupancy in *in vitro* nucleosome (invitrosome) [12] reconstitution experiments, we analyzed the reproducibility of positioning and coverage results between multiple independent invitrosome experiments. In all experiments, invitrosomes were formed by salt dialysis using recombinant histone octamer on linearized plasmid DNA [10]. Four different linearized plasmids with identical backbones, but each harboring a different, unique ∼150 bp sequence (see Materials and Methods) at the same insert site (the kat-group plasmids, p4.1, p4.2, p4.3 and p4.4), were used as the DNA template in separate invitrosome experiments (Figure S1, kat-group backbone).

To allow invitrosomes to form on the DNA templates in positions influenced only by underlying DNA preferences and to eliminate the effects of steric hindrance or positioning by neighboring nucleosomes [6], [14], [15], [16], [17], we used a reconstitution ratio of one histone octamer per 1000 bp of plasmid DNA. After *in vitro* formation, mononucleosomes were isolated by MNase digestion; and DNA from these invitrosome cores, representing their positions on the plasmid DNAs, were isolated as previously described [6], [18]. Invitrosome cores were ligated with barcoded adaptors and sequenced (Tables 1 & 2). A total of 860,741 invitrosome core DNAs were sequenced for these four plasmid reconstitutions representing ∼6,600 to ∼8,200 fold coverage for each experiment.

**Table 1:** Plasmids, Backbones, Primers and Barcodes.

Invitrosome	Source plasmid	Linker pair	Barcode
p4.1	pCR4Blunt-TOPO	AF-SJ-84/AF-SJ-99	CAGT
p4.2	pCR4Blunt-TOPO	AF-SJ-85/AF-SJ-100	GTCT
p4.3	pCR4Blunt-TOPO	AF-SJ-86/AF-SJ-101	TGCT
p4.4	pCR4Blunt-TOPO	AF-SJ-87/AF-SJ-102	CCCT
p7.1	pPD149.40	AF-SJ-88/AF-SJ-103	AACT
p7.2	pPD149.40	AF-SJ-89/AF-SJ-104	GCAT
p7.3	pPD149.40	AF-SJ-90/AF-SJ-105	CGAT
p7.4	pPD149.40	AF-SJ-91/AF-SJ-106	TAAT
p7.5	pPD149.40	AF-SJ-92/AF-SJ-107	ATAT
p7.6	pPD149.40	AF-SJ-93/AF-SJ-108	TCTT
p7.7	pPD149.40	AF-SJ-94/AF-SJ-109	GATT

**Table 2:** Reads for Coverage Plots.

Invitrosome	Raw	Mapped	Filtered
p4.1	230867 (100%)	224453 (97.2%)	217405 (94.2%)
p4.2	230672 (100%)	229591 (99.5%)	225536 (97.8%)
p4.3	185104 (100%)	182461 (98.6%)	179213 (96.8%)
p4.4	214098 (100%)	207059 (96.7%)	204239 (95.4%)
kat-group total	860741 (100%)	843564 (98.0%)	826393 (96.0%)
p7.1	192686 (100%)	191075 (99.2%)	179666 (93.2%)
p7.2	148552 (100%)	142340 (95.8%)	134453 (90.5%)
p7.3	68977 (100%)	65821 (95.4%)	61940 (89.8%)
p7.4	113936 (100%)	109367 (96.0%)	95264 (83.6%)
p7.5	49407 (100%)	48081 (97.3%)	41552 (84.1%)
p7.6p7.6_601	330983 (100%) 330983 (100%)	321480 (97.1%) 321480 (97.1%)	188176 (56.9%) 321480 (97.1%)
p7.7	353261 (100%)	344100 (97.4%)	330525 (93.6%)
sèt-group total*	1257802 (100%)	1222264 (97.2%)	1031576 (82.0%)
ALL	2118543 (100%)	2065828 (97.5%)	1857969 (87.7%)

*excluding p7.6_601.

We parsed our sequence reads into individual experiments according to the embedded barcodes and mapped the reads back to their respective reference plasmids. In order to avoid any end bias [13], [19], [20] or influence from the different ∼150 bp inserts in our plasmids, we filtered our reads such that reads mapping to within 147 bp of either end of the linearized reference plasmid, as well as reads mapping to within 147 bp upstream or downstream of the insertion site or to the insertion site itself, were excluded. Any reads that overlapped these filtered areas or would overlap them when extended to 147 bp were excluded from further analysis. The resulting filtered read sets were used to create coverage plots representing nucleosome occupancy and positioning on the plasmids (Tables 1 & 2).

### Consistency and reproducibility between invitrosome experiments

**Figure 1:**
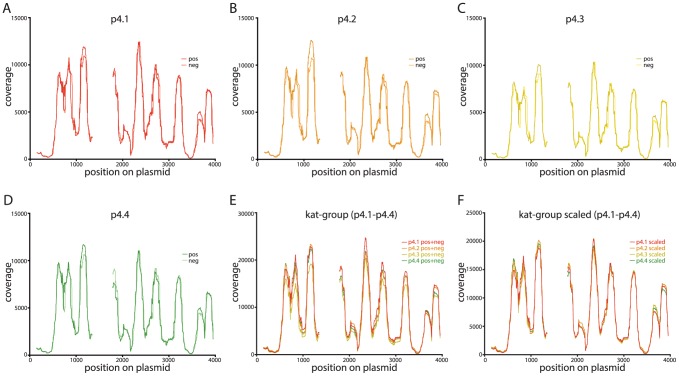
Invitrosome coverage plots for kat-group plasmids show near identical coverage patterns. Invitrosome coverage on each plasmid is plotted on the y-axis and the plasmid coordinates in bp are plotted on the x-axis. Gaps in the plot represent the trimmed insert site and end regions on each plasmid. **1A–1D** Non-scaled coverage plots with forward (pos) and reverse (neg) invitrosomes plotted separately for plasmids p4.1-p4.4. **1E** Non-scaled coverage plots with forward and reverse invitrosomes (pos+neg) combined for each plasmid. All four kat-group plasmids are shown together. **1F** Combined and scaled coverage plots for all four kat-group plasmids where each plot is normalized to the plasmid with the least coverage for direct comparison.

For each of our invitrosome experiments, we generated coverage plots by extending all mapped reads to a total length of 147 bp from the read start site. After this extension, the number of invitrosomes that occupied each site on the plasmid was calculated. Histograms of nucleosome occupancy at each site and for each plasmid, looking at both forward-mapping reads and reverse-mapping reads independently, were generated (Figure 1). Visual inspection and comparison of the forward-read coverage plots to the reverse-read coverage plots and between the plots of all four independent experiments showed striking near identity in their coverage and positioning patterns. For better visual comparison of these plots, we normalized the data between the four experiments by making combined forward- and reverse-read coverage plots for each experiment and then scaling the plots to the lowest coverage plot (by read count) among the four (see Materials and Methods). This allowed visual discrimination and direct comparison of all four experiments on a single plot (Figure 1F), further confirming the striking near identity of the results of all the experiments.

### Invitrosome reproducibility on other DNAs

**Figure 2:**
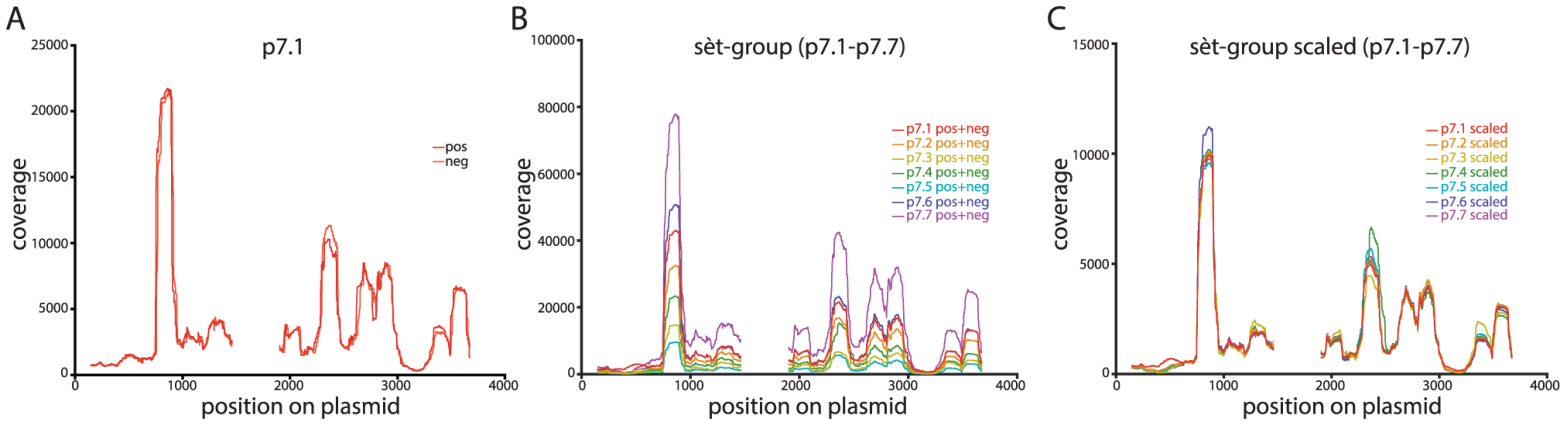
Invitrosome coverage plots for an alternative plasmid backbone (the sèt-group plasmids) also show near identical coverage patterns. **2A** Example coverage plot for one of the sèt-group plasmids (p7.1) with both forward (pos) and reverse (neg) invitrosomes plotted separately on the same graph. **2B** Non-scaled coverage plots with forward and reverse invitrosomes (pos+neg) combined for each of the seven sèt-group plasmids plotted on the same graph. **2C** Combined and scaled coverage plots for all seven sèt -group plasmids, where for direct comparison, each plot is normalized to the plasmid with the least coverage and plotted on the same graph.

One concern is that the high reproducibility of the invitrosome analyses that we observed above is actually an effect unique to the plasmid backbone we used in our experiments. In order to address this question we performed *in vitro* nucleosome reconstitution experiments on a set of seven new plasmids, again all with the same plasmid backbone, but different from the backbone used in our previous experiments (the sèt-group plasmids: p7.1, p7.2, p7.3, p7.4, p7.5, p7.6 and p7.7). Like the previous set of plasmids, each of these seven plasmids harbored a different ∼150 bp sequence at a unique site within the plasmid backbone (Figure S1, sèt-group backbone). These invitrosome experiments were performed with reconstitution, digestion, sequencing and analysis identical to the experiments described above. Like our first set of experiments, this second set of experiments showed extremely high reproducibility between the seven plasmids both with forward- versus reverse-read coverage plots and with combined coverage plots of the seven plasmids (Figure 2 and Figure S2). One notable feature of these data is that, unlike our previous set of experiments, the sequencing read coverage on these seven plasmids varied considerably (Figure 2B). Despite this variation, the visual patterns in the plots were entirely consistent, and after normalization by scaling, showed near identity (Figure 2C). After all coverage plots were scaled to the coverage plot with the least amount of coverage (Figures 1F and 2C), the effective coverages for the kat-group and sèt-group plasmids were 6660X and 1663X respectively.

Notwithstanding this qualitative visual conformation of consistency and reproducibility between invitrosome experiments, we needed a metric to quantify the similarity between our experiments. We chose to use Pearson's correlation coefficients for our metric.

### Quantitative analysis of invitrosome experiment reproducibility

**Figure 3:**
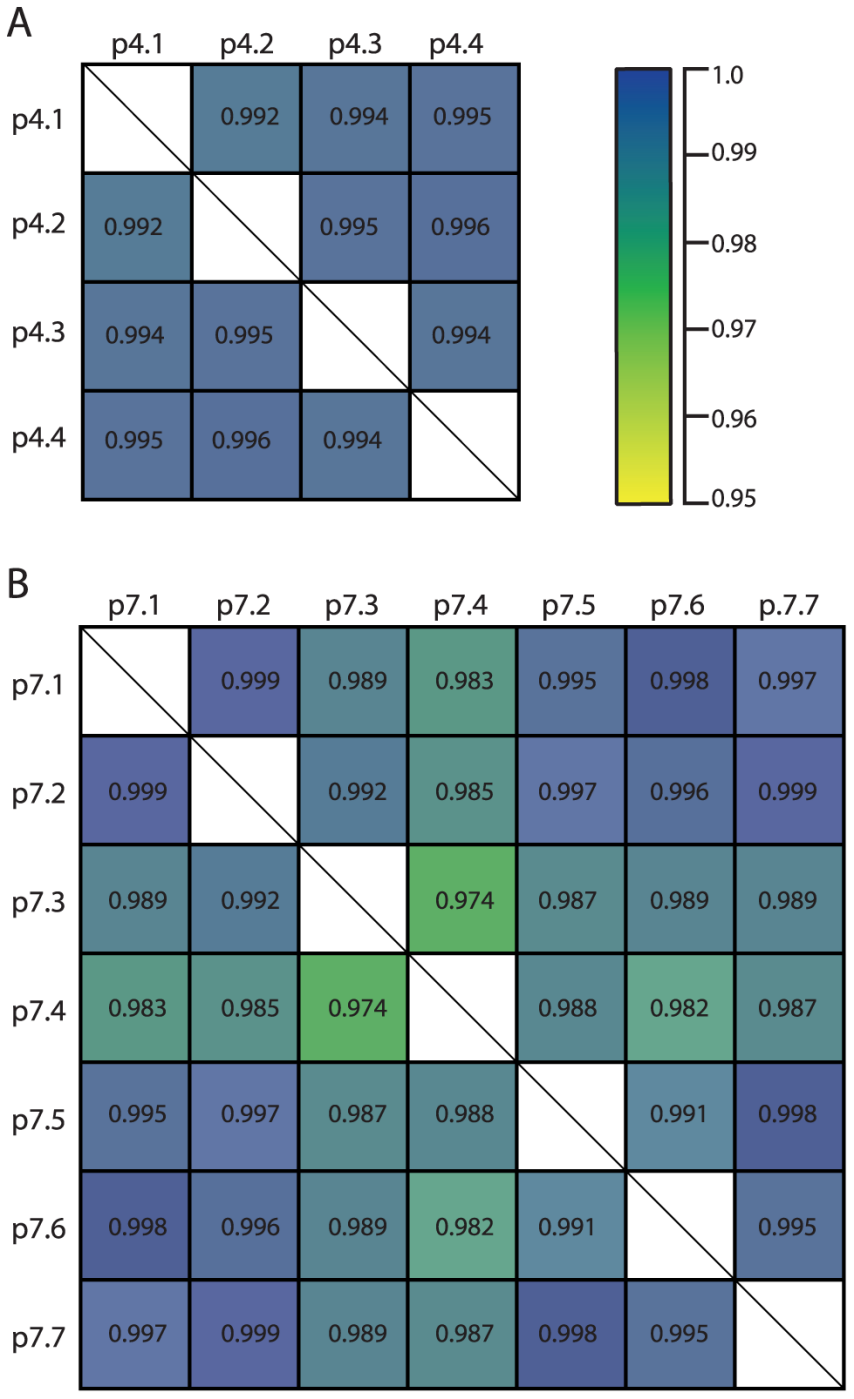
Correlation matrices displaying Pearson's correlation values for scaled coverage plots quantitatively demonstrate high correlations between invitrosome experiments. **3A** Pearson's correlation matrix for coverage plots from plasmids p4.1-p4.4. **3B** Pearson's correlation matrix for coverage plots from plasmids p7.1-p7.7. In both matrices, high to lower correlation values range in color from blue to yellow as show.

We calculated the Pearson's correlation between each pair of coverage plots within each group and made a Pearson's correlation coefficient matrix for the different coverage plots. We were pleased to see extremely high correlations ranging from a low of 0.974 to a near perfect correlation of 0.999 (Figure 3). Thus our qualitative visual and quantitative computational analyses demonstrated the extreme level of reproducibility in our invitrosome experiments. Additionally, visual inspection of the coverage plots, especially in the plots of the sèt-group plasmids, shows not only consistent relative occupancy, but also consistent positioning in individual sites with some very well positioned nucleosomes (Figure 1F and 2C). However, given the extremely high coverage levels of all our individual invitrosome experiments (non-scaled plots ranging from 1,663X to 13,225X), we wanted to know if this observed consistency and reproducibility is only possible between experiments with enormously high coverage like the ones we have here.

### Effect of variable coverage density

**Figure 4:**
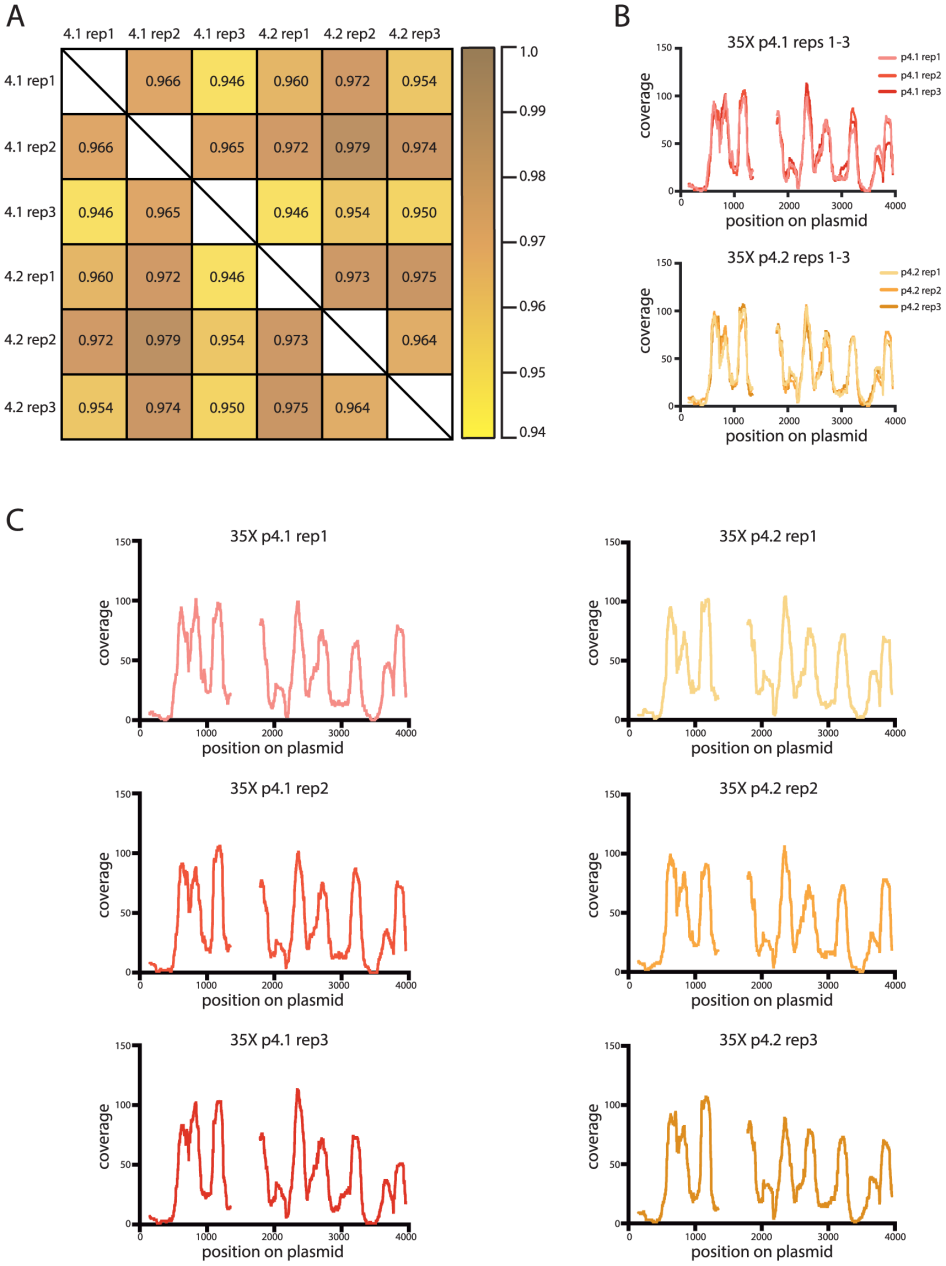
High correlations are maintained at experimentally relevant invitrosome coverage levels. **4A** Pearson's correlation matrix for the coverage plots generated from three replicates at 35X coverage of plasmids p4.1 and p4.2. **4B** (top) Combined coverage plots for replicates 1-3 of plasmid p4.1 at 35X and (bottom) combined coverage plots for replicates 1-3 of plasmid p4.2 at 35X. **4C** All six replicates of the 35X coverage plots. (Left) coverage plots of replicates 1-3 of plasmid p4.1 at 35X. (Right) coverage plots of replicates 1-3 of plasmid p4.2 at 35X.

Our analysis showed that our method for *in vitro* reconstitutions, mononucleosome core DNA isolation, and sequencing yielded very consistent results across samples and experiments when coverage is high. To determine the minimum level of coverage required to achieve similar or minimally acceptable Pearson's correlation coefficients, we randomly extracted different amounts of filtered reads for each plasmid corresponding to the following levels of coverage: 1X, 2.5X, 5X, 10X, 15X, 25X, 35X, 50X, 100X and 500X. We initially chose two plasmids of each backbone type for this analysis: p4.1 and p4.2 for the kat-group plasmids, and p7.1 and p7.2 for the sèt-group plasmids. We performed three replicate read extractions for each of the chosen plasmids at each of the ten coverage levels examined. We generated coverage plots as described above for each replicate at each coverage level and calculated Pearson's correlation coefficient values (Tables S1). Figure 4 is an example of this analysis for one pair of plasmids (p4.1 and p4.2) at one (35X) coverage level.

**Figure 5:**
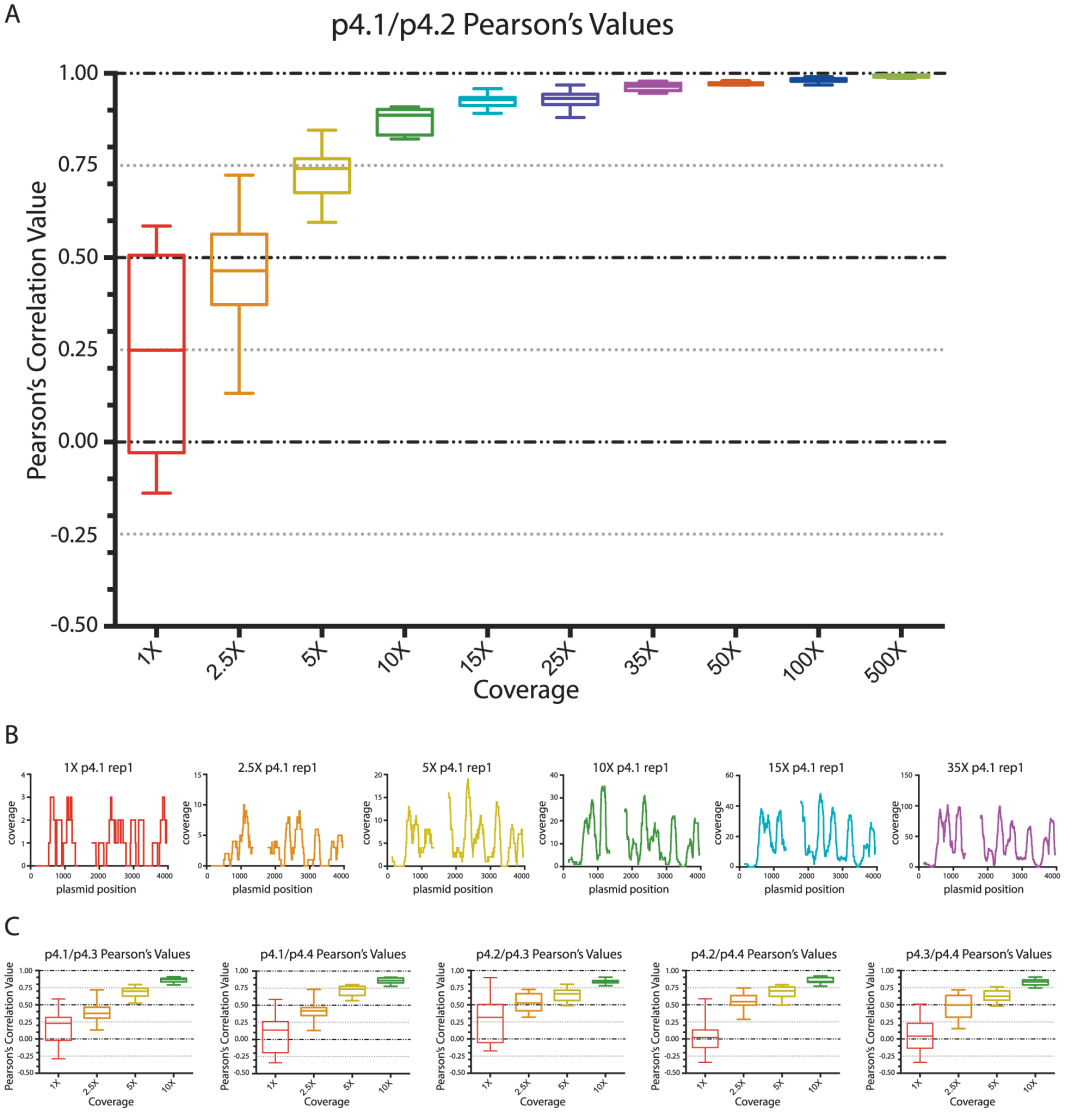
Comparison of correlation values show minimal acceptable coverage levels for invitrosome experiments. **5A** Range of correlation values at each coverage level for the pair-wise comparisons of the three replicates for plasmid p4.1 and the three replicates for plasmid p.4.2. The Pearson's correlation values are on the y-axis and the coverage levels are on the x-axis. The range of correlation values for each coverage level is plotted as a whisker plot composed of the 30 pair-wise comparisons at each coverage level. **5B** Coverage plots for replicate 1 of plasmid p4.1 at coverage levels: 1X, 2.5X, 5X, 10X, 15X and 35X. The colors of the coverage plots correspond to the whisker-plot colors of the same coverage levels in 5A. **5C** Whisker plot graphs showing the variability of correlation values at low levels of coverage (1X-10X) for all combinations of the kat-group plasmids. In 5A–5C the colors red, orange, yellow, green, indigo and purple represent data from coverage levels of 1X, 2.5X, 5X, 10X, 15X and 35X, respectively.

To visually analyze the range of Pearson's correlation coefficient values at each coverage level we plotted the Pearson's correlation coefficients as whisker plots. As expected, Pearson's correlations between plasmid replicates were inconsistent at low coverage levels and became better with increasing coverage (Figure 5A). As can be seen in Figure 5B, once coverage reached 35X, the plasmid backbone-specific pattern observed in the full, normalized-coverage experiments became apparent (Compare Figure 1F and Figure 5B last panel).

**Figure 6:**
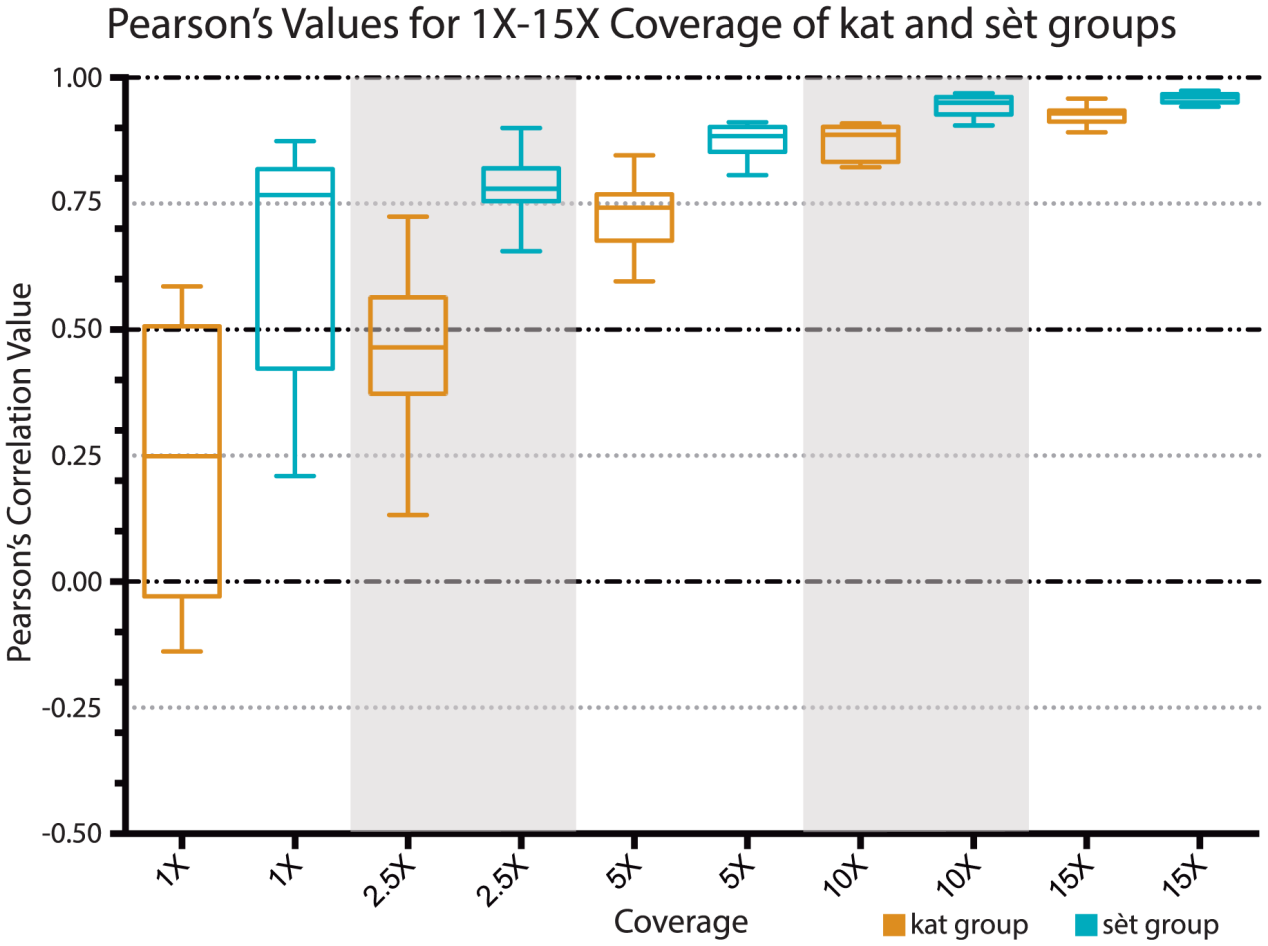
Higher degree of correlation between low-level coverage of sèt-group plasmids compared to low-level coverage of kat-group plasmids. Range of correlation values for coverage levels 1X, 2.5X, 5X, 10X and 15X are displayed as a whisker plot. The pair-wise comparisons of the three replicates for plasmid p4.1 and the three replicates for plasmid p.4.2 from the kat-group plasmids are displayed as orange whisker-boxes and the pair-wise comparisons of the three replicates for plasmid p7.1 and the three replicates for plasmid p.7.2 from the sèt-group plasmids as blue whisker-boxes. The Pearson's correlation values are on the y-axis and the coverage levels are on the x-axis.

Due to the wide range of correlation coefficients observed at the lower coverage levels from replicates of plasmids p4.1 and p4.2, we performed the same analysis on the rest of the kat-group plasmids at the lower coverage levels (1X-10X) and compared them to one another (Figure 5C). We also did this low-coverage-level pair-wise analysis for all of the sèt-group plasmids (Figures S3–S7). As can be seen in Figure 5C, regardless of the plasmid pair, for the kat-group plasmids, correlations at very low coverage levels (1X and 2.5X) are quite variable and extremely low to nonexistent. But surprisingly, at even moderate levels of coverage (5X and 10X), correlations become modestly good (above 0.5 and 0.75 respectively). Interestingly, the Pearson's correlation coefficient values for the sèt-group plasmids at lower coverage levels (1X-10X) are strikingly higher than those of the kat-group plasmids (Figure 6 and Figures S4–S7). The possible cause of this will be discussed below.

## Conclusions

Here we have shown that *in vitro* nucleosome reconstitution experiments are reproducible and highly consistent when read coverage is sufficiently deep. We have quantified correlation coefficients between reconstitutions using Pearson's correlations, and as expected, Pearson's correlation values steadily rise and have less variation as coverage increases, with values reaching as high as 0.999 between experiments. The most dramatic gains in increasing Pearson's correlation values with minimal variation are made once 35X coverage is reached where values are 0.946 and above.

It is important to note that in these experiments we have used nothing but the raw invitrosome reads and their coverage plots to calculate correlation values. In most studies of nucleosome positioning using high-throughput sequencing, large numbers of raw nucleosome reads are mapped and then converted to coverage plots that are used to call individually positioned nucleosome dyads using various probability statistics or smoothing algorithms which greatly decrease the variability within the data *e.g.* [5], [21]. The resulting data are then used to compare results between experiments resulting in higher correlations than if the raw data were used without such manipulation. Here, by using only the raw data we have not artificially increased our correlation values, but shown that such manipulations are not necessary to achieve even near-perfect reproducibility between invitrosome experiments using our experimental conditions.

Additionally, we found that the correlations between low coverage experiments are quite variable and at least somewhat dependent on the plasmid backbone. Specifically, the Pearson's correlation values for the kat-group plasmids at low coverage (1-10X) were strikingly lower than the values for the sèt-group plasmids (Figure 6). We believe that this is due to intrinsic differences in the backbones. The coverage plots for the kat-group plasmids have several more peaks representing positioned nucleosomes than those for the sèt-group plasmids (Figures 1F and 2C), indicating that the kat-group backbone has more places where nucleosomes are likely to form and *in toto* result in a more uniform occupancy across the entire plasmid backbone; whereas a plurality if not the majority of nucleosomes in the sèt-group plasmid backbones occupy two or three specific sites with one of these sites being very highly occupied. We believe this to be the cause of the higher correlation values at low coverage levels for the sèt-group plasmids; with more nucleosomes in fewer sites there is less possible variation even at low levels of coverage. This is most likely due to the inherent higher affinity of these few sites. Thus when a nucleosome forms on a sèt-group plasmid backbone, it is likely to form in one of a few specific sites rather than one of the many possible sites on a kat-group plasmid backbone. Many nucleosomes in a few sites give a better correlation than the same number of nucleosomes spread over many sites.

**Figure 7:**
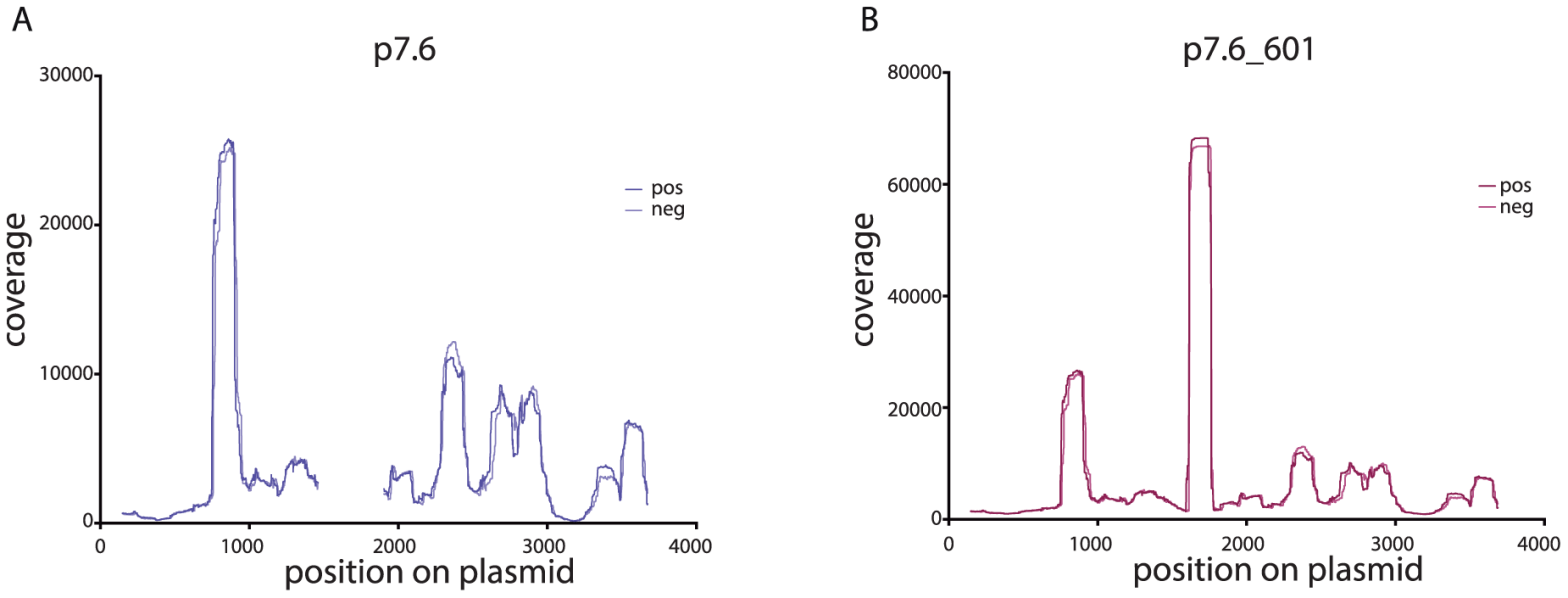
The 601 nucleosome-positioning sequence positions a plurality of invitrosomes. **7A** Coverage plot for the sèt-group plasmid p7.6 with both forward (pos) and reverse (neg) invitrosomes plotted separately on the same graph. **7B** Coverage plot for plasmid p7.6_601 which harbors the 601 nucleosome-positioning sequence with both forward (pos) and reverse (neg) invitrosomes plotted separately on the same graph. The highest peak starting at about base pair 1600 is where the 601 sequence begins.

**Figure 8:**
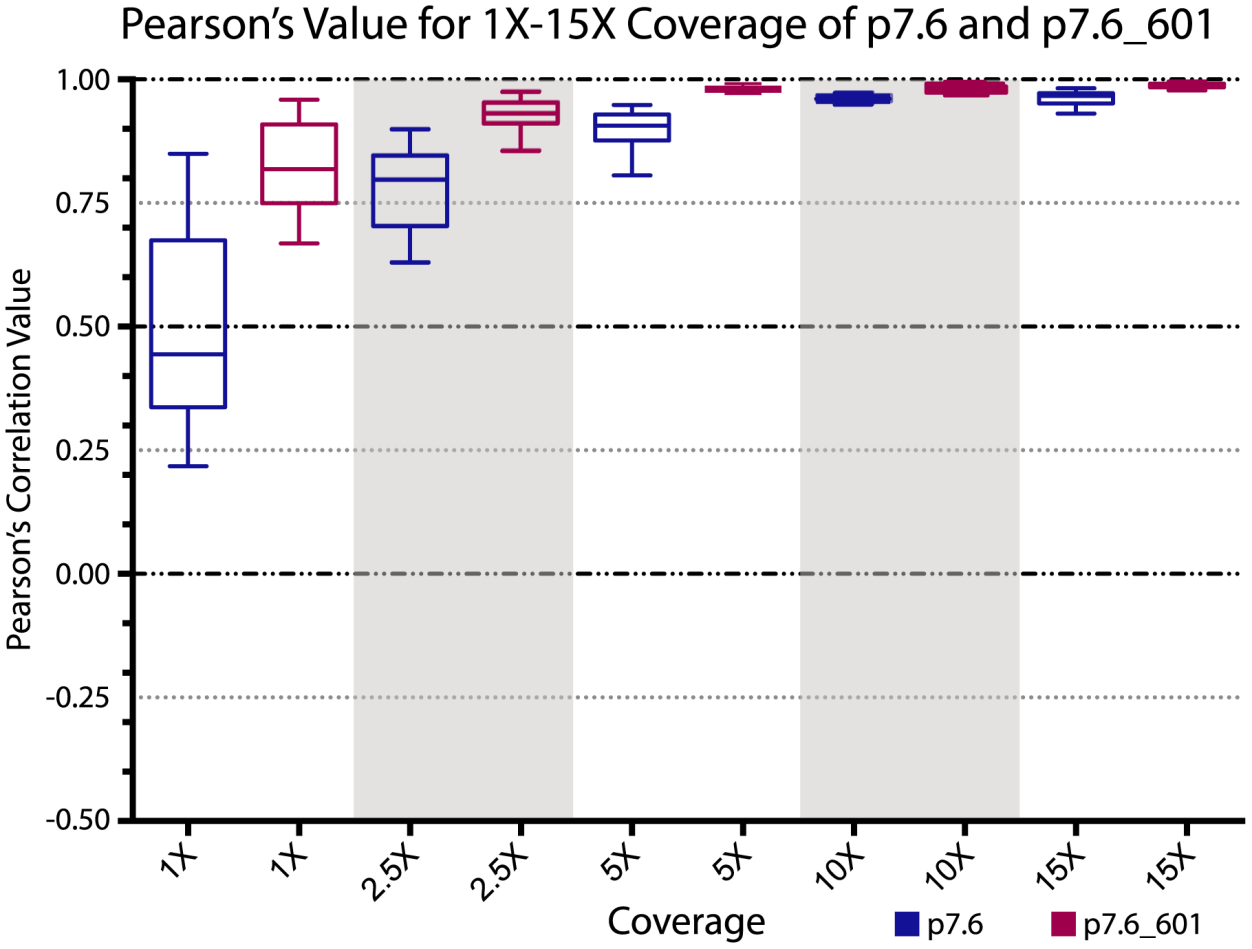
Higher degree of correlation between coverage of p7.6_601 compared to coverage of p7.6. Range of correlation values for coverage levels 1X, 2.5X, 5X, 10X and 15X are displayed as a whisker plot. The correlation values from the pair-wise comparisons of the six replicates for plasmid p7.6 and the six replicates for plasmid p.7.6_601 are displayed as blue whisker boxes and purple whisker boxes respectively. The Pearson's correlation values are on the y-axis and the coverage levels are on the x-axis.

This hypothesis can be easily tested by embedding a known strong nucleosome-positioning sequence into one of our plasmid backbones and verifying that adding such a highly attractive nucleosome-positioning sequence reduces the coverage depth required to obtain good correlation values. The unique ∼150 bp insert sequence in the sèt-group plasmid p7.6 is actually the 601 nucleosome positioning sequence. The 601 sequence is the highest affinity DNA sequence known that causes occupancy and positioning of nucleosomes in *in vitro* nucleosome reconstitutions [11]. To test if the addition of the 601 sequence would result in better correlation values at lower coverage levels as hypothesized, we performed six replicate random read extractions for both the p7.6 plasmid and the p7.6 plasmid with the 601 sequence (p7.6_601). In the case of p7.6_601 we now included in the results the reads that mapped to the 601 insert site and its flanking regions that had previously been excluded from our analyses. These replicate read extractions were done for both plasmid data sets at 1X, 2.5X, 5X, 10X and 15X coverage levels. We generated coverage plots as described above for each replicate at each coverage level and calculated Pearson's correlation coefficient values between the six replicates of p7.6 and separately between the six replicates of p7.6_601 (Tables S1). Full-read coverage plots for both p7.6 and p7.6_601 demonstrate that the 601 sequence is indeed highly attractive to nucleosome formation (Figure 7) and, that with the addition of the 601 sequence, a plurality if not a majority of reads now map to the 601 site (Figure 7B). As seen by whisker plots of the ranges of correlation coefficient values at each coverage level, Pearson's correlations between the p7.6_601 plasmid replicates were much higher than the Pearson's correlations between the p7.6 plasmid replicates at all coverage levels (Figure 8). As can be seen in Figure 8, surprisingly good correlations are achieved at even the 1X coverage depth in the p7.6_601 plasmid, supporting our hypothesis.

The above explanation is illustrative of why the general criticisms of the reproducibility of *in vitro* nucleosome reconstitution experiments are not valid. Having multiple possible nucleosome formation sites on a given piece of DNA, and seeing these multiple outputs in invitrosome experiments, does not show that these experiments are inconsistent, but that they just have not been performed and analyzed at sufficient depth. As demonstrated by our extremely-high-coverage experiments, and even our moderate-coverage experiments, the preference of invitrosome formation in such experiments is very consistent, and on defined stretches of DNA, is even limited to an easily quantifiable number of possible positions.

Thus we propose that studies looking at nucleosome positioning using *in vitro* reconstitution should ideally try to achieve a 35X coverage of the target genome or locus in order to have maximum confidence in the results, but also recognize that significant correlations are seen at levels as low as 5X coverage and should be used as an absolute minimum. In extreme cases (*i.e.*, p7.6_601), even 1X coverage results in a satisfactory correlation (Figure 8). Given the current levels of output using next-generation sequencing technologies these target coverages are easily achievable and quite reasonable.

In these experiments we have used MNase digestion of invitrosomes and high throughput sequencing of mononucleosome DNA fragment ends as our output to define individual nucleosome positions and overall nucleosome coverage. This analysis relies heavily on the patterns that are reveled by MNase digestion and assumes, as have many previous studies [6], [7], [9], that such digestions along with their known and unknown biases are representative of *in vitro* nucleosome positions. It is possible that the striking consistency between our experiments is a result of our particular technique and conditions, but this in no way detracts from our conclusions about the reproducibility of such experiments. It should be emphasized that, as exemplified by the differences in correlation values at various coverage depths between the kat-group plasmids, the sèt-group plasmids and p7.6_601 (Figures 6 and 8), the coverage depth necessary to achieve acceptable correlation values is at least dependent on the nature of the DNA sequence, and probably also dependent on the specific system used, and will vary with other factors such as octamer to DNA ratio and reconstitution method and conditions. Further analyses using other probes and conditions to reveal *in vitro* nucleosome positions and DNA preferences, and analysis of the consistency between these techniques will be an exciting avenue for future exploration.

## Materials and Methods

### Reconstitution of *in vitro* nucleosomes (invitrosomes)

The 11 different plasmids used in these experiments were derived from two plasmid backbones. The four kat-group plasmids were all derived from the pCR4Blunt-TOPO plasmid (Life Technologies) and each had a different ∼150 bp cloned insert in its cloning site. The seven sèt-group plasmids were derived from the pPD149.40 plasmid (a gift from Andrew Fire and the Fire Lab) (Data pPD149.40 S1) and had different ∼150 bp cloned inserts in their unique *Avr* II restriction sites. The ∼150 bp inserts in the plasmids were various putative nucleosome- positioning or repelling sequences that were designed for another analysis, and thus were masked and excluded from these analyses as to not obfuscate our testing of the reproducibility of invitrosome experiments, except in the case of p7.6_601 where the insert and flanking sequences were retained as described. For *in vitro* nucleosome reconstitution, all of the plasmids were linearized by restriction digestion with *Sca* I, cutting, in both groups, at a unique *Sca* I restriction site opposite of the insert sites. Invitrosomes were formed on the *Sca* I-linearized plasmid templates in separate experiments using the previously described salt dialysis technique [10]. Recombinant *Xenopus* histone octamers (a gift from Geeta Narlikar and the Narlikar Lab) and DNA templates were reconstituted at a ratio of 1 octamer per 1000 bp of linear plasmid DNA, resulting in a 1:4 molar ratio of DNA to histone octamer. Specifically, for each template 9.67 ug of DNA and 1.50 ug of histone octamer were reconstituted in a total volume of 200 ul.

### Isolation of invitrosome core DNA fragments

Invitrosome core DNAs from all 11 invitrosome reconstitutions were isolated as previously described [6], [18]. Briefly, for each experiment 60 ul of invitrosomes were digested with MNase (Roche) at 1 U/ul for 15 min at room temperature, histone proteins were digested using proteinase K (Roche), DNA was isolated using phenol/chloroform extractions and ethanol precipitation, mononucleosome core DNAs were isolated on a 2% UltraPure Agarose (Life Technologies) gel, extracted using a QIAquick Gel Extraction Kit (Qiagen), and eluted in 30 ul of EB (Qiagen).

### End repair, linker ligation and library sequencing

Invitrosome core DNAs were processed and ligated with sequencing adaptors as previously described [6] with the following exceptions. For all samples the entire 30 ul of isolated invitrosome DNA cores were processed. Previously annealed duplex barcoded adaptors were added to each sample according to Table 1 (Data Adaptors S1 for adaptor sequences) and were incubated with T4 DNA ligase for 4.5 hours rather than 6.5 hours. After the ligated bands were isolated there was no amplification of the libraries, but rather 12 ul (out of 30 ul) of each of the 11 barcoded libraries were pooled together to make a single multiplexed Illumina library. This multiplexed library was sequenced on a single lane of the Illumina GAII system resulting in 2,118,543 single-end, 36-bp reads corresponding to the 11 plasmids (see Table 2 and Data Raw Reads S1).

### Nucleosome Mapping

Multiplexed reads were parsed by barcode using custom Perl scripts. After removal of the 4-bp barcodes, the 32-bp parsed reads were mapped back to their respective reference plasmids using a local installation of BLASTN. The BLASTN settings used were –task blastn –best_hit_overhang .1. Reads were analyzed with Fred Tan's custom Perl script summaryPsl-v2.pl [2], and for each read with multiple hits in the BLASTN output, only the hit with the best bit-score was chosen and used in our analysis.

### Nucleosome Coverage

Coverage plots were created with custom Perl scripts by informatically extending upstream (from reverse reads) or downstream (from forward reads) a nucleosome length of 147 bp from the start of the read. Every bp within the reference DNAs was given a count of 1 for each nucleosome overlapping that site. Counts were compiled and used to create a coverage plot. Combined coverage plots were made by adding the counts at identical positions from corresponding positive and negative coverage plots. In order to eliminate any positional effects due to end bias or the putative positioning sequences, BLASTN outputs went through additional filters to remove all reads that overlapped a 147 bp window on either end of the linearized plasmid and flanking the insert (except in the case of p7.6_601 where reads mapping to the insert and flanking regions were retained). These pools of filtered reads were used as inputs for extracting reads to achieve a specific coverage level.

### Scaling Coverage plots

To normalize coverage plots with unequal coverage levels, all coverage plots were scaled to the plot with the lowest level of coverage before performing Pearson's correlations. Scalars were calculated by dividing the number of filtered reads (Table2 reads for coverage plots) for a coverage plot by the number of filtered reads for the coverage plot with the least coverage. The value for the coverage plot at each base pair was then divided by this scalar to yield normalized, scaled coverage plots.

### Pearson's correlation coefficients

Pearson's correlation coefficients were calculated using Prism 6 version 6.0d for Mac OSX. Only values that were not in the insert and 147 bp filtering window were used in the calculations, except in the case of p7.6_601 as described. If there were no counts at a specific bp, its value was left blank.

## Supporting Information

Figure S1 **Diagram of plasmid backbones.** Linear depiction of both the kat-group plasmid and the sèt-group plasmid backbones. The size of the plasmids is indicated in bp by the scale bar at the bottom and the areas excluded or “trimmed” from the analysis are shown in gold. The variable ∼150 bp inserts in the different plasmids are shown by the cross-hatched shading within the central trimmed regions.
(EPS)

Figure S2 **Invitrosome coverage plots for sèt-group plasmids show near identical coverage patterns.** Invitrosome coverage on each plasmid is plotted on the y-axis and the plasmid coordinates in bp are plotted on the x-axis. Gaps in the plot represent the trimmed insert site and end regions on each plasmid. **1A–1F** Non-scaled coverage plots with forward (pos) and reverse (neg) invitrosomes plotted separately for plasmids p7.2-p7.7.
(EPS)

Figure S3 **Whisker plots for the sèt-group plasmids.** Range of correlation values at each coverage level for the pair-wise comparisons of the three replicates for plasmid p7.1 and the three replicates for plasmid p.7.2. The Pearson's correlation values are on the y-axis and the coverage levels are on the x-axis. The range of correlation values for each coverage level is plotted as a whisker plot composed of the 30 pair-wise comparisons at each coverage level.
(EPS)

Figure S4 Whisker plot graphs showing the variability of correlation values at low levels of coverage (1X-10X) for all combinations of the sèt -group plasmids. The Pearson's correlation values are on the y-axis and the coverage levels are on the x-axis. The range of correlation values for each coverage level is plotted as a whisker plot composed of the 30 pair-wise comparisons at each coverage level.
(EPS)

Figure S5 Whisker plot graphs showing the variability of correlation values at low levels of coverage (1X-10X) for all combinations of the sèt -group plasmids. The Pearson's correlation values are on the y-axis and the coverage levels are on the x-axis. The range of correlation values for each coverage level is plotted as a whisker plot composed of the 30 pair-wise comparisons at each coverage level.
(EPS)

Figure S6 Whisker plot graphs showing the variability of correlation values at low levels of coverage (1X-10X) for all combinations of the sèt -group plasmids. The Pearson's correlation values are on the y-axis and the coverage levels are on the x-axis. The range of correlation values for each coverage level is plotted as a whisker plot composed of the 30 pair-wise comparisons at each coverage level.
(EPS)

Figure S7 Whisker plot graphs showing the variability of correlation values at low levels of coverage (1X-10X) for all combinations of the sèt -group plasmids. The Pearson's correlation values are on the y-axis and the coverage levels are on the x-axis. The range of correlation values for each coverage level is plotted as a whisker plot composed of the 30 pair-wise comparisons at each coverage level.
(EPS)

Tables S1 **Excel file of Pearson's Correlation Coefficient matrices for all experiments.**
(XLSX)

Data pPD149.40 S1 **The sequence of plasmid pPD149.40.**
(TXT)

Data Adapters S1 **Adapter sequences used in the study.**
(DOC)

Data Raw Reads S1 **Raw Illumina sequencing reads used in this analysis.**
(ZIP)
