# Supplementary material for: Reproducibility and Consistency of In Vitro Nucleosome Reconstitutions Demonstrated by Invitrosome Isolation and Sequencing
Source: PLoS One. 2014 Aug 5;9(8):e103752. doi: 10.1371/journal.pone.0103752 (PMC4122377; doi:10.1371/journal.pone.0103752)
Supplement: Data Adapters S1 — Adapter sequences used in the study. (DOC) [file pone.0103752.s010.doc]

Adaptor Sequences (barcodes are in blue for forward and red for reverse ologos)

AF-SJ-84 5’-AATGATACGGCGACCACCGAGATCTACACTCTTTCCCTACACGACGCTCTTCCGATCTCAGT

AF-SJ-85 5’-AATGATACGGCGACCACCGAGATCTACACTCTTTCCCTACACGACGCTCTTCCGATCTGTCT

AF-SJ-86 5’-AATGATACGGCGACCACCGAGATCTACACTCTTTCCCTACACGACGCTCTTCCGATCTTGCT

AF-SJ-87 5’-AATGATACGGCGACCACCGAGATCTACACTCTTTCCCTACACGACGCTCTTCCGATCTCCCT

AF-SJ-88 5’-AATGATACGGCGACCACCGAGATCTACACTCTTTCCCTACACGACGCTCTTCCGATCTAACT

AF-SJ-89 5’-AATGATACGGCGACCACCGAGATCTACACTCTTTCCCTACACGACGCTCTTCCGATCTGCAT

AF-SJ-90 5’-AATGATACGGCGACCACCGAGATCTACACTCTTTCCCTACACGACGCTCTTCCGATCTCGAT

AF-SJ-91 5’-AATGATACGGCGACCACCGAGATCTACACTCTTTCCCTACACGACGCTCTTCCGATCTTAAT

AF-SJ-92 5’-AATGATACGGCGACCACCGAGATCTACACTCTTTCCCTACACGACGCTCTTCCGATCTATAT

AF-SJ-93 5’-AATGATACGGCGACCACCGAGATCTACACTCTTTCCCTACACGACGCTCTTCCGATCTTCTT

AF-SJ-94 5’-AATGATACGGCGACCACCGAGATCTACACTCTTTCCCTACACGACGCTCTTCCGATCTGATT

AF-SJ-99 5’P-CTGAGATCGGAAGTCGTATGCCGTCTTCTGCTTG

AF-SJ-100 5’P-GACAGATCGGAAGTCGTATGCCGTCTTCTGCTTG

AF-SJ-101 5’P-GCAAGATCGGAAGTCGTATGCCGTCTTCTGCTTG

AF-SJ-102 5’P-GGGAGATCGGAAGTCGTATGCCGTCTTCTGCTTG

AF-SJ-103 5’P-GTTAGATCGGAAGTCGTATGCCGTCTTCTGCTTG

AF-SJ-104 5’P-TGCAGATCGGAAGTCGTATGCCGTCTTCTGCTTG

AF-SJ-105 5’P-TCGAGATCGGAAGTCGTATGCCGTCTTCTGCTTG

AF-SJ-106 5’P-TTAAGATCGGAAGTCGTATGCCGTCTTCTGCTTG

AF-SJ-107 5’P-TATAGATCGGAAGTCGTATGCCGTCTTCTGCTTG

AF-SJ-108 5’P-AGAAGATCGGAAGTCGTATGCCGTCTTCTGCTTG

AF-SJ-109 5’P-ATCAGATCGGAAGTCGTATGCCGTCTTCTGCTTG
